# Account of a Case of Pulmonary Consumption, in Which Nearly the Whole of the Right Lung Was Converted into an Immense Vomica, Attended with Universal Adhesion, and Partial Absorption of the Pleural Sac

**Published:** 1834-04-01

**Authors:** William Stroud

**Affiliations:** Physician to the Northern Dispensary, &c.


*Account of a Case of Pulmonary Consumption, in which nearly the whole of the right lung was converted into an immense vomica, attended with universal adhesion, and partial absorption of the pleural sac.* Communicated to the Harveian Society, by William Stroud, m.d., Physician to the Northern Dispensary, &c.

On the 31st of March, 1832, I was requested to visit, as a patient of the Northern Dispensary, James T***y, aged twenty-six years, and formerly a domestic servant. I found him labouring under a severe pectoral complaint, of four months' continuance, which had been preceded by diarrhœa, and was ascribed to his having passed the two previous months in a damp house. He has a distressing, and convulsive cough, which disturbs sleep, and excites pain about the sternum, the head, and the inner sides of the thighs. His expectoration is very thick, and glutinous, but not copious. He is generally in bed, and feels best when lying down. On assuming the erect posture, the cough is aggravated; and, by an effort between coughing and vomiting, a yellow, puriform liquid is expectorated. Formerly he could not lie well on the right side, but can now lie with ease on either side, as well as on the back. The chest is much emaciated, and the ribs are conspicuous. In the right side, which is rather hollow, a pulsation can be perceived, on listening with attention; but it yields little sound on percussion, and no respiratory murmur, except under the clavicle. The left side is more resonant on percussion; and, except at its lower part, yields a tolerable respiratory sound. The action of the heart is moderate, the pulse at the wrist, in the recumbent posture, is one hundred, and feeble. He is very weak, and thin, has lately had at intervals several rigors, but has little fever, and no pain, except in the front of the chest, on coughing. A short time since, he was somewhat jaundiced; and, about a month ago, a quantity of viscid, blackish mucus, accompanied with tormina, was discharged from the bowels. The dejections, at the same time, were dark-coloured, but afterwards became yellowish, and the urine, which is not yet quite natural, was red, and turbid. His tongue is whitish in the middle, with red edges, he has little thirst, or appetite, and occasionally vomits his food. The abdomen bears pressure well, and exhibits no morbid appearance. About two years before his present complaint, he laboured for six weeks under continued fever, but without any marked affection of the chest; and his relatives are said not to be subject to phthisis.

*Prescriptions.* Aquæ puræ, f ℥vj.; Tinct. Iodini, f ʒss.; Tinct. Camphor. comp., Tinct. Gentian. comp., *aa* f ʒiv., Sumatur f ℥j. ter in dies. Extract. Hyoscyam. gr. v. omni vespere. Pectori superiori imponatur Emplastr. Picis.

During the following four, or five weeks, he underwent little change; but, on the whole, seemed to improve. On account of the confined state of his bowels, purgative medicine was occasionally administered. As the mixture was thought to excite a sense of heat in the chest, the Tinct. Iodin. was reduced one half, and the Tinct. Hyoscyam. was given, instead of the Tinct. Camphor. comp. With a view to procure rest at night, the Pil. Sapon. cum Opio were substituted for the Extract. Hyoscyam., but without much success.

May 10th. I found him dressed, and sitting in another room, where he had been taking his supper, and, in appearance, better than I ever saw him, either before, or afterwards. His cough is mitigated; his expectoration is whiter, thinner, and more frothy; his bowels are open, without medicine; and his sleep is less disturbed. His pulse, at the same time, is 120 in the sitting posture, and weak; his respiration is scarcely audible, except at the upper part of the left side of the chest, and his general frame is much emaciated.

*Prescriptions.* Extract. Aloes, Extract. Jalap. *aa* gr. xv.; Pulv. Gambog. gr. v.; Pulv. Colocynth. gr. x.; Ol. Croti gutt. v.; Ol. Juniperi, q. s. Fiant pil. xij. quarum sumantur ij. primo mane, prout opus fuerit. Aquæ puræ, f ℥vj.; Potass. Nitrat. ʒj.; Tinct. Iodin. m. xx.; Tinct. Hyoscyam. f ʒiss.; Tinct. Gent. comp. f ʒiv. Sumatur f ℥j. ter in dies. Sumat omni vespere Extract. Hyoscyam. gr. v. Bibat in dies Decoct. Lichen. Oct. j.

19th. Almost immediately after the last report, an unfavourable change took place. The cough, having again become violent, disturbs his sleep, and excites severe pain in the right side of the chest, which now yields a tolerable sound on percussion, but no respiratory murmur. To this side the patient refers the whole of his disease; and from thence, alone, supposes the expectoration, which is now copious, frothy, and mucilaginous, to proceed. The pulse in the recumbent posture is 116, and weak. The right cheek is sometimes coloured, while the left is pale; and, after a fit of coughing, the hand, and other parts on the right side of the body, are often warm, while those on the opposite side are cool.

*Prescriptions.* Pectori dextro superiori affigantur hirud.

vj. Aquæ ferventis, Oct. j.; Gummi Tragacanth. ʒj. Fiat potio mucilaginosa, cui frigefactæ adjiciantur Tinct. Hyoscyam. f iij.; Vin. Ant. Tart. f ʒiij. Sumatur f ℥j. ter in dies. Sumat. omni vespere Pil. Sapon. cum Opio, gr. v. Repetr. Pil. Cathart. et Decoct. Lichen.

31st. The leeches drew a good deal of dark-coloured blood, with temporary relief; but a violent cough, which is much excited on swallowing liquids, has renewed the pain on the right side of the chest. The expectoration, which is less copious, resembles in appearance thick mucilage, and contains whitish particles. During the last two, or three days, he has suffered from diarrhœa, with flatulence, and is in consequence weaker, and more emaciated; yet the pulse in the recumbent posture is only 106, and tolerably strong, but the countenance is pale, hollow, and almost Hippocratic.

*Prescriptions.* Mist. Cretæ, f ℥vj.; Tinct. Digital. m. xx.; Tinct. Camphor. comp. f ʒiv. Sumatur f ℥j. ter in dies. Repetr. Pil. Sapon. cum Opio, et Decoct. Lichen.

June 2d. The bowels are more composed; the countenance is improved, and he is rather better; but the cough, which is excited whenever he rises in bed, or swallows any liquid, is still distressing. The skin of the chest is hot. Although the right side is hollowed, or contracted in the middle, the ribs are protuberant, with deep intervening furrows. On applying the ear to this side, a metallic tinkling is now, for the first time, perceived.

*Prescriptions.* Pectori dextro admoveatur Emplastr. Cantharid. Rep. med.

8th. The blister operated well, and gave some relief; but there still remains a dull pain in the right side of the chest, which is aggravated by the erect posture, and attended with a copious expectoration.

*Prescriptions.* Aquæ puræ, f ℥vj.; Potass. Nitrat. ʒj.; Pulv. Acaciæ Gummi, ʒiij.; Tinct. Digital. f ʒss.; Tinct. Camphor. comp. f ʒiv. Sumatur f ℥j. ter in dies. Repetr. Pil. Sapon. cum Opio, et Decoct. Lichen.

10th. He is somewhat better. The right side of the chest is now nearly free from pain, but very sensitive to pressure. The Pil. Sapon. cum Opio have little effect in procuring sleep; and the draught seems to excite cough, which is always aggravated by the erect, and relieved by the recumbent posture. The expectoration is copious, and mucilaginous. His pulse is 100, and sufficiently full, and strong: his tongue is red, with good appetite, and no thirst; his bowels, and urine, are natural. The middle of the right side of the chest, which is hollow, or depressed, now yields a clear sound on percussion, and, when the ear is applied, a shrill tinkling, like that produced by water dropping from a height into a metallic capsule. The lower part of this side is extuberant, and without resonance. The respiratory murmur is heard on the left side only, and there, chiefly, at the upper end, near the clavicle.

*Prescriptions.* Potion. Mucilag. antea præscript. f℥vj.; Tinct. Digital. fʒss.; Tinct. Camphor. comp. f ʒiv. Sumatur f℥j. ter in dies. Repetr. Pil. Sapon. cum Opio, et Decoct. Lichen.

14th. Yesterday the diarrhœa returned; the appetite for food ceased; and, although the cough is somewhat relieved, he is now evidently, but gradually, sinking.

*Prescriptions.* Mist. Cretæ f℥vj.; Tinct. Opii, m. xx.; Tinct. Cinchon. comp. f ʒiv. Sumatur f℥j. ter in dies. Repetr. Pil. Sapon. cum Opio, et Decoct. Lichen.

21st. Although the Tinct. Catechu was substituted for the Tinct. Cinchon. comp., the diarrhœa continued, and increased. The dejections were numerous, and yellow, and were attended with pain about the umbilicus, and with a sense of pulsation in the lower part of the abdomen. The urine was natural. On getting out of bed yesterday, the right side of the chest was attacked with acute pain, and with a peculiar, and distressing feeling, as of instant death, which occasioned him to cry out. At length, about seven this morning, after some tossing of the limbs, and slight convulsive movements, in full consciousness of his approaching dissolution, he slowly, and tranquilly expired, nearly three months from the period of my first visit. After death, the whole right side, together with the right leg, was found somewhat contracted, so that the body could not be laid out straight.

*Post-mortem Appearances.* With the assistance of Mr. G. Harwood, I obtained an inspection two days afterwards, June 24, 1832, when the following appearances were observed.

*General Conditions.* The body was exceedingly emaciated, the bones being everywhere very conspicuous; yet, owing in part, no doubt, to the great care which had been taken of the young man, there was neither sloughing, nor ulceration. The face was hollow, and wasted; the eyes, and mouth were half open; but the hair, which was of a dark colour, was thick, and strong. The right side of the chest was considerably contracted, as if flattened, or compressed; the ribs being bent at a sharp angle on the outer side, and hollow, or collapsed in front. Both sides of the chest sounded tolerably well on percussion, but the left better than the right.

The body contained scarcely any blood, and no fat. The commencement of putrescence was announced, in the abdomen, by a slight degree of greenness, fetor, and emphysema. The skin was so extremely emaciated, and adherent to the subjacent parts, as to require much pains to dissect it off. The muscles, although wasted, were tough.

The head, and spine were not examined.

*Thorax.* The principal morbid appearances were those of pulmonary consumption, by which the greater part of the right lung had been converted into an immense vomica, closely adhering to the parietes of the chest. The pericardium, which was rather ample, and loose, as if it had once enclosed a larger heart, exhibited more serous effusion, and of a deeper colour than usual. The heart was rather small, but firm, and sound; and, notwithstanding the general weakness, and wasting, the walls of the left ventricle were of considerable thickness, and strength. In the cavities of each side was a moderate quantity of blood, chiefly in the state of soft, red coagulum; and, in those of the left side, there was also a yellowish, semitransparent coagulum, owing apparently, to the slowness with which death had taken place.

The mediastinum was thick, short, and diffuse. The left pleural sac was free from adhesions, and contained a small quantity of serous liquid. The left lung did not fill its cavity; yet, when extracted, was found to be firm, heavy, and voluminous, owing to an extensive deposit of tubercular matter, the hardness, and irregularity of which could be felt externally, on pinching the surface. Of this deposit, a small portion was in the state of minute grains, of equal size, and of globular shape. A larger portion had formed numerous vomicæ, from the size of a pea, to that of a walnut. Some of them, situated near the surface, had no other exterior covering than the pleura, which exhibited, in consequence, corresponding dimples, or depressions. Several of them, lying in clusters, were confluent, or, at least, continuous. They were, in general, half filled with a thin, puriform liquid, and, in some instances, were lined by false membrane. But, by far the greater part of the tubercular deposit was in the intermediate state of opaque, whitish, flocculent masses, continuous at the centre, but broken at the circumference, and, in texture, or configuration, much resembling certain varieties of granite. There was little appearance of interstitial inflammation: on the contrary, several lobules, in immediate contact with the tuberculated portions, seemed to be quite healthy, and capable of performing their function. The trachea, and its primary branches, were spacious, and the lining membrane was pale. Some of the larger bronchi contained a little white, opaque, frothy liquid, but this was not generally diffused; and, on making sections into the lung, there was no effusion either of blood, serum, or mucus. The bronchial glands were enlarged. Some of them were reddish, and others of their usual dark colour.

The greater part of the right lung was, as before stated, converted into an immense, and nearly empty vomica, capable of holding three, or four pints, but actually containing only a small portion of thin, puriform liquid. The remaining pulmonary substance was hepatized, and of a dark grey colour, forming a dilated sac, of which the thickest side was towards the mediastinum, and the thinner adhered closely, and universally, to the parietes of the chest; the two pleuræ being, as it were, amalgamated, and inseparable. In some places, the united membrane, and probably the periosteum, also, were entirely removed by absorption, down to the ribs; several of the lowermost of which had, consequently, produced, near their angles, small, white, exostoses, partly smooth, partly rough, and foliated, like morbid epiphyses, attached by an intervening membrane to the original bone. The internal surface of this spacious pulmonary cavern was irregularly roughened by coarse granulations, and lined with thick, yellow pus. It was traversed, more especially at the upper end, by several fleshy ropes, apparently, the insulated remains of blood-vessels, which had resisted the general ulceration. Its extent downwards, or towards the abdomen, was much limited, so that the diaphragm rose within the chest nearly as high as the fifth rib. The communication of this cavity with the bronchial tubes was not conspicuous; but, from its nearly empty state, and from the quality, and amount of the preceding expectoration, the existence of such a communication might fairly be inferred.

*Abdomen.* The peritoneal sac contained little, or no effused liquid. The omentum was voluminous, dark-coloured, and entirely destitute of fat; and, near the navel, adhered firmly to the parietes by a slender chord. The stomach, which was distended with gas, was very large, thin, and capable, seemingly, of holding three, or four pints; its capacity, in part, perhaps, depending on the assiduity with which the young man had been supplied with nourishment by his attendants. Its mucous membrane was sound, presenting only a small extent of petechiated surface, and a few rugæ. The pylorus was thin, and its aperture large. The small intestines were generally plump, and somewhat distended with liquid, and gas. Some portions, comprehending their entire cylinder, were of a deep red colour, but without any thickening, contraction, or adhesion. The duodenum, like the stomach, was capacious, and inflated.

The large intestine, except from the sigmoid flexure downwards, was contracted. The cœcum was narrow, and scarcely projected beyond the end of the ileum. The appendix vermiformis was short, serpentine, and adherent. The mucous membrane of the colon, lined with yellow, healthy-looking feculent matter, was partially inflamed, and presented numerous small ulcers, of a round, or oval form. Some of these ulcers were seated on an inflamed base; but others appeared as if punched out of the sound membrane by a sharp cutting instrument. A few similar ulcers were found on the inner surface of the extremity of the ileum, and on the edges of the ileo-colic valve, but did not seem to extend further upwards in the small intestines. The veins of the mesentery were, in general, much distended with blood. Its glands were enlarged, oblong, and of a dirty-white colour. Near the cœcum, a few smaller glands were harder, and more globular.

The liver was of fair size. Its peritoneal coat was rather dark coloured; owing, perhaps, to the incipient extrication of sulphuretted hydrogen gas. Internally, it was soft, and slightly mottled throughout, with angular, or polygonal, white lines; but, on section, it yielded no exudation, either of blood, bile, or serum; although the venous trunks traversing its substance, as well as the inferior cava, were large, and thin. The gall-bladder contained a little orange-coloured bile, diluted with mucus, but without concretions. The spleen was rather small, and conglomerate, its internal condition varying in different parts; for, on making sections into it, one portion was found of its usual dark hue, while an adjacent portion was of a light red colour, and soft texture, with a paler, peritoneal coat. The pancreas was small, slender, and soft, with distinct, and full-sized lobules. The kidneys, containing little urine, and destitute of fat, were fleshy, firm, and nearly in a natural state; but the right kidney, corresponding to the pulmonary vomica, was diminutive, and its ureter very slender. The urinary bladder was empty, and collapsed.

Remarks. The interest of this case of pulmonary consumption chiefly consists in the contrast which it presents between the progress of the same disease in the opposite sides of the chest; and in the gradual, and silent manner, in which almost the whole of the right lung had been destroyed, and removed; while the left lung, although greatly disorganised, was nearly in a solid state. The whole probably depended on the absence, or, at least, on the low degree of inflammatory action; a circumstance strongly marked in the remaining lung, wherein there was neither adhesion, effusion, nor hepatization, but, on the contrary, small portions of healthy pulmonary substance were, in many instances, intermingled, and in close contact with tubercular masses; showing that inflammation has no necessary connexion with phthisis, either as cause, or as effect. The same fact was, in some measure, displayed in the mucous membrane of the colon, which contained a number of small, circular, phagedænic ulcers, entirely free from surrounding redness, or turgescence; and which were, more probably, the result of nervous, than of vascular action. The irritation of various portions of the mucous lining of the ileum, and colon, so common in the latter stage of phthisis, is, evidently, a sympathetic affection, remotely excited by the diseased lung; and the medium of intercourse in this, and, in other cases, seems to be furnished by the minute, but important, and universally diffused nerves of the ganglionic system, which preside over the nutritive functions; and, according to their varied condition, are capable of increasing, lowering, or modifying vital action, in the parts which they supply, and, thereby, of producing all the diversities of vascular action, as well as of organic composition, or dissolution.

Another interesting circumstance presented by this case is the clearness of its diagnosis, resulting, chiefly, from the absence of respiratory murmur throughout the whole right side of the chest, in conjunction with its contracted, and excavated form, and with deficiency of resonance on percussion, until the immense vomica which it contained had been partially emptied; when, together with returning resonance, the characteristic sign of metallic tinkling was distinctly added. The value of the acoustic signs, on such occasions, may be estimated by comparing the prompt, and certain knowledge of internal conditions, attainable by their aid, with the vague conjecture, or total ignorance, which are the unavoidable consequence of neglecting them.

This case, when compared with the preceding one, displays, also, the striking contrast which exists between the two principal forms of pectoral abscess, namely empyema, and pulmonary vomica, according to their situation, within, or without the pleural sac, in their influence on the dimensions of the thoracic cavity; which the latter tends to diminish, and the former, at least in the first instance, to enlarge. The enlargement is manifestly occasioned by the pressure of a confined, and accumulating liquid. The diminution seems to depend on a slow, but powerful contractile process, which, in the pleura, as in other serous membranes, whether original, or adventitious, is induced by inflammation; and which, as my friend, Dr.

Hodgkin, and other pathologists have shown, usually produces a remarkable effect on the shape, and size, of the parts to which they are attached.

In the last place, this case, in common with many others, illustrates the specific, and, to a certain extent, the hopeless aspect of phthisis, as resulting from a peculiar aberration of the nutritive functions, originating, probably, in the ganglionic system, and, in a great measure, beyond the reach both of investigation, and of relief. In its earliest observable stage, the disease exhibits a number of membranous vesicles, of a distinct, and malignant character, somewhat like obstructed, and dilated follicles; absorbing nutriment from the surrounding parts by their external surface, and secreting within their cavities a whitish, albuminous pulp, at first semitransparent, but afterwards opaque, and, in appearance, resembling soft cheese. Near the surface, and, more especially, at the summit of the lungs, a few tubercles of large size are sometimes found, and may be compared to the indolent deposits which, in the common cellular membrane, give rise to strumous abscesses; but, in the interior of the organ, they are usually small, and numerous. When they have attained a certain period of development, either from the distention of their accumulating contents, or from some other cause, they seem to lose their vitality, become softened, and are commonly discharged into the bronchial tubes, leaving so many empty, and, in general, suppurating cavities in their room.

This process includes all that is essential to phthisis; which, as in the case now under consideration, sometimes pursues a simple and tranquil course, attended with little pain, or disturbance, until, by compression, and interstitial absorption, the greater part of the lungs has been silently removed; so that, when the tubercular growth which has usurped its place falls, at length, into dissolution, the sooner, perhaps, in consequence of being thus by its own agency deprived of nutriment, a vomica of the largest size is suddenly created. If, on the other hand, the tubercular deposit is more limited in its extent, and more equally intermixed with the pulmonary substance by which it is sustained, it may for a considerable time retain its firmness, and the vomicæ in which it ultimately terminates are either small, and detached; or, if larger, attain their bulk, by successive confluence, producing, often, an intricate and extensive labyrinth of pulmonary caverns.

The more active and distressing varieties of phthisis may, perhaps, in part depend on original differences of organization, and of susceptibility; but, more immediately, on the addition of inflammation, in various forms, and degrees, either of the pleura, of the bronchial membrane, or of the pulmonary substance, to the original tubercular deposit. By such inflammation, and its results, the local symptoms of pain, dyspnœa, cough, and expectoration are aggravated, and the organic nerves derived from the eighth pair, and from the ganglionic system, are violently irritated, giving rise to sympathetic disorder in various points, together with hectic fever, cerebral disturbance, and universal restlessness, and distress. In the former case, the greater part of the lungs is gradually, and insidiously undermined, and death is chiefly induced by the abolition of their indispensable function. In the latter, while a large portion of the organ remains apparently sound, the more penetrating inflammation, and ulceration of a small fraction of their substance is sufficient to occasion intense suffering, and to accelerate death, through the medium of fever, and of irritation.

Hence, the supreme importance of early diagnosis, to recognize the morbid disposition, and of early treatment, to anticipate, if possible, the tubercular formation. The hopeless aspect of the complaint, when far advanced, must be evident, from a consideration of its nature, and tendency. Individual cases may, undoubtedly, differ greatly, in severity, and in extent; much may be done by preventive measures; and, more especially, by the cautious use of iodine, the universal antidote of scrofula; pulmonary ulcers, when of limited character, may admit of being cicatrized, or of being lined with false membrane; but, when the disease has once attained a certain growth, no remedy with which we are at present acquainted, or which we are likely to discover, can repair the injury committed, remove with safety the adventitious deposit, or restore the pulmonary substance already destroyed.

